# The Pharmacopœia of the United States of America

**Published:** 1842-10

**Authors:** 


### Art. X.

*The Pharmacopœia of the United States of America.* By Authority of the National Medical Convention, held at Washington, A.D. 1840.—*Philadelphia*, 1842. 8vo, pp. 279.

In the year 1818 the New York Medical Society, at the suggestion of Dr. Lyman Spalding, made a proposition to form a National Pharmacopœia, (the want of which in the United States had long been felt as a serious evil,) under the authority and by the conjoint labours of the profession throughout the Union. The proposal seems to have been received with almost universal approbation. Accordingly, delegates from the different medical bodies assembled in each of the four districts into which the United States were divided, and by them draughts of a Pharmacopœia were prepared, and subsequently submitted to a General Convention which met at Washington on the 1st of January, 1820. The different draughts underwent deliberate examination, and were incorporated into one work, which was adopted with the title of *The Pharmacopœia of the United States of America,* the publication of which was intrusted to a Committee appointed for that purpose. The labours of the Convention did not cease here. It made a provision for the decennial revision of the work; and resolved that the General Convention should be held at Washington in 1830. In accordance with this resolution it assembled on the 4th of January in that year, and after adopting measures for the publication of a revised edition of the Pharmacopœia, which appeared in 1831, it was determined that the third meeting of the Convention should take place in 1840. It accordingly met at Washington on the 1st of January in that year, and appointed a Committee of seven physicians (including Drs. Wood, Bache, and Dunglison,) who, we believe, were the acting members for the revision and publication of the Pharmacopœia. It was not until near the end of the year 1841 that this Committee had completed the review of the work, which had then to be arranged and prepared for the press.

The first two editions of the United States Pharmacopœia were printed in duplicate, namely, in both Latin and English. The publication of a national pharmacopœia in the vernacular language was at that time a novelty; but the Convention felt so strongly the propriety and value of such a proceeding that it boldly, and, as we think, very wisely, resolved to break through the long-established custom. But several reasons, one of which was that other languages than the English were in extensive use in some parts of the United States, led the Convention to publish their Pharmacopœia in both the Latin and English languages conjointly, the Latin being used on the left-hand page, and the corresponding English on the right. The reasons assigned for the duplication of the work do not appear to have had much weight with the Convention at its third meeting, for we find that it resolved to print this revised edition exclusively in English. In the preface to this work it is observed that—

"There seems to be no practical advantage to counterbalance the inconvenience of attempting to present ideas in a language which has no appropriate words to express them, and the labour and expense incurred in printing twice as much matter as is necessary to convey the meaning intended. The recent publication, moreover, of the French Codex and the Edinburgh Pharmacopœia in the vernacular languages of the two countries respectively, gives the sanction of high authority to the course now pursued." (Pref. xvi.)

The general plan and execution of the United States Pharmacopœia are very similar to those of the British ones. But the subdivision of the Materia Medica into a primary and secondary list is peculiar to the former. We cannot say that we are convinced of the advantage or propriety of this proceeding, to which several objections may be urged.

Willdenow's edition of Linnæus's *Catalogus Specierum Plantarum,* and De Candolle's *Prodromus Systematis Naturalis* are the works usually relied on as authorities for the names of plants, while Cuvier's *Règne Animale* is taken as authority for the names of animals. On several occasions, however, other authorities have been cited; though not so frequently, we think, as ought to have been done. Thus the retention of the old Linnæan names of *Laurus Camphora* and *Laurus Sassafras* appears to us peculiarly objectionable, since for the cinnamon plant, which belongs to the same family, Nees's authority has been followed, and his nomenclature adopted. For the sake of consistency and uniformity, therefore, we think the names applied by this distinguished botanist to the other officinal species of *Lauraceæ* ought to have been adopted. We observe that *cinnamon* is declared to be " the bark of Cinnamomum Zeylanicum

(*Nees*) and of Cinnamomum aromaticum (*Nees*)." But though *cinnamon* properly so called is the bark of Nees's Cinnamomum Zeylanicum, his Cinnamonium aromaticum yields *cassia lignea.* So that by this error the United States Pharmacopœia authorises the substitutions of cassia lignea for cinnamon, and likewise the oil of cassia for the genuine oil of cinnamon. In a medicinal point of view this is a matter of little or no importance; but the commercial value of the substances being very different, the Pharmacopœia ought not to have authorised the substitution. Moreover, the proceeding just referred to must lead to great confusion.

We highly approve of the plan adopted in the Prussian, London, and Edinburgh Pharmacopœias, and followed in the present work, of annexing to some of the articles of the Materia Medica and certain preparations, "brief notes indicating the readiest means of ascertaining their genuineness and purity." These notes are, for the most part, very judiciously drawn up; though, in some few instances, they are unnecessarily long, as in the case of *Sulphate of Soda.* We select the note to *Citric Acid* as a fair example of this part of the work, conjoining, for the sake of comparison, the notes respectively given to the same substance by the London and Edinburgh Pharmacopœias:

| *United States Pharmacopœia.* | *London Pharmacopœia.* | *Edinburgh Pharmacopœia.* |
|---|---|---|
| In colourless crystals, wholly dissipated by a red heat, and freely soluble in water. The solution affords with acetate of lead a precipitate soluble in nitric acid, and yields no precipitate when added in excess to a solution of carbonate of potassa. | This acid is soluble in water; what is precipitated from the solution by acetate of lead is dissolved by nitric acid. No salt of potash except the tartrate is precipitated by solution of nitric acid. It is totally dissipated in the fire. | A solution in four parts of water is not precipitated by carbonate of potash: when incinerated with the aid of oxide of mercury no ash is left, or a mere trace. |

Of these three notes we prefer for its conciseness and precision that of the Edinburgh College.—We have been greatly surprised to find no notes attached to *Elaterium, Scammony, Opium,* and many other important articles of the Materia Medica. Surely some means should be pointed out of recognising the gross adulterations practised on elaterium and scammony, and of ascertaining the value of opium.

It is stated that *Acetate of Lead* "is dissolved by distilled water, with a slight turbidness, which is removed by the addition of distilled vinegar." This statement, however, does not apply to *pure* acetate of lead, which yields a perfectly transparent aqueous solution, but to the common sugar of lead of the shops.

Under the head of *Vinegar* we are told that "one fluid ounce is saturated by about thirty-five grains of crystallized bicarbonate of potassa. It affords no precipitate with solution of chloride of barium, and is not coloured by sulphohydric acid." According to this statement one fluid ounce of American vinegar (which is prepared from cider), contains 17·6732 grs. of anhydrous acetic acid; whereas the same amount of the best British malt vinegar, called *proof* or *No.* 24 *vinegar,* contains 20·548 grs. of acid. Moreover, British malt vinegar and French wine vinegar invariably contain a certain quantity of sulphuric acid; indeed, British manufactures are permitted to add $\frac{1}{1000}$ part of sulphuric acid to their vinegar. But if it be true that American vinegar "affords no precipitate with solution of chloride of barium," it is evident that it must be quite free from every trace of sulphuric acid.

In this edition an improvement has been made in the names applied to certain parts of plants used in medicine. Thus on various occasions the terms *rhizoma* and *cormus* (applied to certain underground stems) have been substituted for *radix* and *bulbus*; while in several instances the word *seed* has been correctly altered to *fruit*. Thus the "rhizoma of Acorus Calamus," the "cormus of Colchicum autumnale," and the "fruit of Pimpinella Anisum," are examples in point. In some few instances, however, this principle of giving the right name to parts has not been strictly adhered to, as in the case of the fruit of conium maculatum, which is incorrectly denominated the "seeds."

Many of the processes of the last edition of the American Pharmacopœia have been left with little or no alteration. On several occasions those of the last editions of the London and Edinburgh Pharmacopœias have been adopted, and we are told in the preface that whenever the Committee of revision

"Could wholly approve the processes of the London or Edinburgh College [they] have adopted them in preference to others, from a disposition to promote uniformity in the preparation of medicines, so far as practicable, throughout those countries in which the English language is used."

The committee have followed the Edinburgh college in permitting most tinctures to be prepared either by the old method of digestion, or by the modern and much more expeditious process of percolation or displacement, as introduced by the French chemists. General directions for the performance of the process, which however requires no slight skill and experience to become successful, are given in both the Edinburgh and United States Pharmacopœias.

It would be tedious, we apprehend, to enter into a detailed account of the different preparations contained in the American Pharmacopœia. A few, however, deserve notice, in consequence of not being contained in the British pharmacopœias.

"*Vinum Ergotæ. Wine of Ergot.* Take of ergot, bruised, two ounces; wine a pint (wine measure). Macerate for fourteen days, with occasional agitation; then express, and filter through paper." (p. 241.)

It is obvious that every fluid ounce of this preparation contains one drachm of ergot.

"*Acidum Tannicum. Tannic Acid.* (*Tannin.*) Take of galls, in powder, sulphuric ether, each a sufficient quantity. Put into a glass adapter, loosely closed at its lower cord with carded cotton, sufficient powdered galls to fill about one-half of it, and press the powder slightly. Then fit the adapter accurately to the mouth of a receiving vessel, fill it with the sulphuric ether, and close the upper orifice so as to prevent the escape of the ether by evaporation. The liquid which passes separates into two unequal portions, of which the lower is much smaller in quantity and much denser than the upper. When the ether ceases to pass, pour fresh portions upon the galls, until the lower stratum of liquid in the receiver no longer increases. Then separate this from the upper, put it into a capsule and evaporate with a moderate heat to dryness. Lastly, rub what remains into powder.

"The upper portion of liquid will yield by distillation a quantity of ether, which, when washed with water, may be employed in a subsequent operation." (p. 62.)

Tannic acid occasionally proves an exceedingly useful astringent in positive hemorrhages and in profuse discharges from the mucous membranes. It may also be used as an antidote in poisoning by the vegetable alkalies. The dose of it is three or more grains.

"*Unguentum Mezerei. Ointment of Mezereon.* Take of mezereon, sliced transversely, four ounces; lard fourteen ounces; white wax two ounces. Moisten the mezereon with a little alcohol and beat it in an iron mortar until reduced to a fibrous mass; then digest it with the lard, in a salt-water bath for twelve hours, strain with strong expression, and allow the strained liquid to cool slowly, so that any undissolved matters may subside. From these separate the medicated lard, melt it with the wax at a moderate heat, and stir them constantly until they are cold." (p. 233.)

This is the *Pommade épispastique au Garou* of the French Codex. It is an irritating application, which may be used as an epispastic or as a substitute for savine ointment in maintaining a discharge from blistered surfaces.

In concluding our brief notice of the Pharmacopœia of the United States, we beg to express our opinion that it is highly creditable to the knowledge, industry, and talents of the gentlemen composing the committee of revision, and to whom the getting up of the work was intrusted. It is obvious that great care has been bestowed on its preparation, as well as on its passage through the press. The alterations have been very judiciously effected, and all such new medicines and preparations introduced as the progress of discovery in the healing art has rendered necessary. As we have in some few instances expressed our dissent from the statements contained in this work, we would remind our readers that experience has fully shown that every modern pharmacopœia, British and Continental as well as American, presents numerous topics adapted for the cavillings of critics; and we verily believe that the present work contains fewer of these than many others of its kind produced much nearer home.

---

